# Mobile and Wearable Technology for the Monitoring of Diabetes-Related Parameters: Systematic Review

**DOI:** 10.2196/25138

**Published:** 2021-06-03

**Authors:** Ciro Rodriguez-León, Claudia Villalonga, Manuel Munoz-Torres, Jonatan R Ruiz, Oresti Banos

**Affiliations:** 1 Research Center for Information and Communication Technologies University of Granada Granada Spain; 2 Department of Computer Science University of Cienfuegos Cienfuegos Cuba; 3 Departament of Medicine University of Granada Granada Spain; 4 Endocrinology and Nutrition Unit Hospital Universitario Clinico San Cecilio Granada Spain; 5 Centro de Investigación Biomédica en Red sobre Fragilidad y Envejecimiento Saludable Instituto de Salud Carlos III Madrid Spain; 6 PROmoting FITness and Health through Physical Activity Research Group Department of Physical Education and Sports University of Granada Granada Spain

**Keywords:** diabetes, monitoring, passive sensing, smartphone, wearable, mobile phone

## Abstract

**Background:**

Diabetes mellitus is a metabolic disorder that affects hundreds of millions of people worldwide and causes several million deaths every year. Such a dramatic scenario puts some pressure on administrations, care services, and the scientific community to seek novel solutions that may help control and deal effectively with this condition and its consequences.

**Objective:**

This study aims to review the literature on the use of modern mobile and wearable technology for monitoring parameters that condition the development or evolution of diabetes mellitus.

**Methods:**

A systematic review of articles published between January 2010 and July 2020 was performed according to the PRISMA (Preferred Reporting Items for Systematic Reviews and Meta-Analyses) guidelines. Manuscripts were identified through searching the databases Web of Science, Scopus, and PubMed as well as through hand searching. Manuscripts were included if they involved the measurement of diabetes-related parameters such as blood glucose level, performed physical activity, or feet condition via wearable or mobile devices. The quality of the included studies was assessed using the Newcastle-Ottawa Scale.

**Results:**

The search yielded 1981 articles. A total of 26 publications met the eligibility criteria and were included in the review. Studies predominantly used wearable devices to monitor diabetes-related parameters. The accelerometer was by far the most used sensor, followed by the glucose monitor and heart rate monitor. Most studies applied some type of processing to the collected data, mainly consisting of statistical analysis or machine learning for activity recognition, finding associations among health outcomes, and diagnosing conditions related to diabetes. Few studies have focused on type 2 diabetes, even when this is the most prevalent type and the only preventable one. None of the studies focused on common diabetes complications. Clinical trials were fairly limited or nonexistent in most of the studies, with a common lack of detail about cohorts and case selection, comparability, and outcomes. Explicit endorsement by ethics committees or review boards was missing in most studies. Privacy or security issues were seldom addressed, and even if they were addressed, they were addressed at a rather insufficient level.

**Conclusions:**

The use of mobile and wearable devices for the monitoring of diabetes-related parameters shows early promise. Its development can benefit patients with diabetes, health care professionals, and researchers. However, this field is still in its early stages. Future work must pay special attention to *privacy and security* issues, the use of new emerging sensor technologies, the combination of mobile and clinical data, and the development of validated clinical trials.

## Introduction

### Background

Diabetes mellitus (DM) is a metabolic disorder primarily characterized by high blood glucose levels (GLs). People with DM are more likely to have other major health problems. Therefore, the chances for them to require special medical attention increases as the patients’ quality of life decreases [1]. Statistics from the International Diabetes Federation show that 463 million people had DM worldwide in 2019, with a total of 4.2 million estimated deaths that year. The projection of these data is alarming for the next years, and by 2045, an increase of 33.9% is estimated; thus, 700 million people would have DM [2]. Such a continued increase in the prevalence of DM is mainly justified by the global rise in obesity, driven foremost by people’s unwholesome lifestyles and urbanization [3]. According to the International Diabetes Federation, more people have died from DM than from other diseases sometimes categorized as more dangerous or receiving more attention from health agencies or governments [4].

DM is normally categorized into 3 groups: type 1 diabetes (T1D), type 2 diabetes (T2D), and gestational diabetes (GD). T1D affects between 5% and 10% of patients with DM and most often occurs in young people [5,6]. T1D is fundamentally characterized by a severe problem of insulin secretion. Patients are required to use an external source of insulin to balance their blood GLs. These multiple daily doses can be administered through injections or continuous insulin pumps. T2D affects between 90% and 95% of patients with DM, usually adults and senior citizens [5,6]. In this case, the pathophysiological mechanism is insulin resistance, and over time, the body loses the ability to secrete the right amount of this hormone. These type of patients with DM have several options to treat their condition.

Irrespective of the type of DM, low or too high GLs in the blood for long periods can induce several complications in patients, leading to premature death in worst cases. Critical hypoglycemia can cause comatose states and induce seizures. Chronic hyperglycemia can cause vascular damage; affect the heart, kidneys, eyes, and nerves; and lead to other serious complications [7,8]. These complications of DM can be classified into 2 types: microvascular (related to retinopathies, nephropathies, and neuropathies) and macrovascular (mainly related to cardiovascular problems) [9,10].

Extensive tests have proven that appropriate metabolic control in all DM types can delay the onset and evolution of its complications [10]. In addition, early diagnosis, continuous health care, and adequate self-monitoring of the disease by patients are key for preventing or minimizing complications. Moreover, several studies indicate that T2D can be prevented by maintaining a healthy lifestyle, attaining adequate nutrition, performing physical activity, and avoiding obesity [11]. Therefore, some parameters are of special interest to be monitored by patients with DM, such as body weight, GL, performed physical activity, blood pressure, low-density lipoprotein cholesterol, triglycerides, microalbuminuria, glycated hemoglobin level [12], acquired calories, feet condition, eye conditions, and stress levels, among others [4,13]. For all issues related to DM, governments and institutions must urgently implement new strategies, primarily fostered by the potential of diabetes technologies, to decrease the risk factors that lead to T2D and guarantee quality health care for people with DM [14].

The health community uses the term *diabetes technology* for devices and software that patients with DM use to aid their condition. According to this classification, diabetes technology has 2 main categories: insulin administration and blood glucose monitoring. Insulin pumps are the most popular devices used for insulin administration. Continuous glucose monitors (CGMs) are most often used for monitoring blood GLs. In recent years, hybrid devices have been developed, including both functions. When this technology is used properly, it can improve the quality of life of patients. However, the complexity and rapid changes in this field can be an obstacle to their widespread use by patients [15]. Although diabetes technologies have been mostly dominated by this type of devices, it is only recently that the advances in mobile and wearable technologies have burst in to complement prior diabetes technology with a new generation of digital solutions at the reach of most people.

The widespread adoption of wearable and mobile technologies around the world offers new opportunities for researchers to provide medical care and information in a portable and affordable way [16]. Smartphones stand out because of their strong computational features and pervasiveness. As of 2019, 2.5 billion people owned smartphones [17], which is far beyond the number of desktops or laptop computer ownership in countries such as the United States [18]. Even among older adults, smartphone ownership has doubled. By the beginning of 2019, there were approximately 350.4 million people using wearable technology [19], namely, devices worn directly on or loosely attached to a person [20], with a growing trend in the use of wrist-worn devices [21]. This technology has distinctive applications in the health care field because of its capacity to gather, store, and transmit data and sometimes even process it. Both patients and physicians can leverage these features for the management, treatment, and assessment of their conditions. Despite the aforementioned capabilities of mobile and wearable technology, these devices are not yet extensively used in clinical settings [20].

Several sensors are readily available on regular smartphones, such as the accelerometer (ACC), GPS, camera, ambient light, and microphone, among others. The data collected by these sensors can be used to determine the user context [22]. For example, physical activity or calories burned by the user can normally be measured using a smartphone’s motion sensors (ACC and gyroscope) [23]. Wearables have features that increasingly compare to those of regular smartphones, including, in some cases, built-in GPS, barometer, heart rate (HR), ACC, or gyroscope sensors. In addition, wearables outmatch smartphones while sensing physiological signs, such as HR, electrocardiogram (ECG), or skin temperature, which are considered of particular interest for the monitoring of DM-related parameters. Some of these physiological measurement capabilities can also be instrumented in smartphones via external pluggable devices, although only for occasional use [24]. By processing the collected data generated by the sensors on smartphones and wearables, it is possible to monitor many of the relevant parameters for patients with DM, such as GL, blood pressure, calories, physical activity, feet condition, eye condition, and stress levels. In addition, one of the most relevant characteristics of this technology is its capacity to monitor in a continuous, passive, and unobtrusive way, without necessarily interfering with people’s regular daily living.

Mobile and wearable devices generate an enormous amount of data, and their ability to process these data is beyond human skills [25]. This is why sophisticated mechanisms such as artificial intelligence (AI) are most often used in combination with these devices to digest and extract meaningful knowledge from the gathered data. AI is widely used to support advanced analytics and provide individualized medical assistance [26]. In addition, a growing number of health care companies are applying AI algorithms to discover relevant clinical information from large amounts of data [27]. The main reasons for this growth include the explosive increase in the amount of data available, along with the improved performance of intelligent methodologies capable of handling and processing it. AI is also attracting great attention to DM, as the amount of data acquired electronically by patients with DM has grown. Proper management of these large volumes of data is expected to increase the quality of life of patients with DM [28]. Thus, AI may play a key role in the recognition of these systems as routine therapeutic aids for patients with DM.

### Objective

Although several manuscripts have been published on the use of mobile and wearable technology for monitoring parameters that condition the development and evolution of DM, hereafter monitoring of DM-related parameters, this subject has not been systematically reviewed to the best of the authors’ knowledge. Therefore, the goal of this study is to review the published literature on the use of mobile and wearable technology for the monitoring of DM-related parameters. Three specific research questions are defined to guide this study: (1) How are DM-related parameters studied using mobile and wearable technology? (2) How are the devices and sensors used to monitor DM-related parameters? and (3) What processing is given to the collected mobile and wearable data?

## Methods

### Overview

The PRISMA (Preferred Reporting Items for Systematic Reviews and Meta-Analyses) guidelines [29] were followed to perform a systematic review of the literature on mobile and wearable sensing for the monitoring of DM-related parameters. Moreover, the Newcastle-Ottawa Scale (NOS) [30] was used to assess the quality of the studies. The specific methodology followed is described in the following sections.

### Information Source

Studies were identified by searching electronic databases and scanning publications from a reference list of authors. The search was performed using 3 reference web-based citation databases: Web of Science (WoS), Scopus, and PubMed. The last search was performed on July 28, 2020. The queries used for the database search are listed in Textbox 1. The terms used in the queries and the combination thereof aim to match the title, abstract, or keywords of the manuscripts. In addition, we handsearched on ResearchGate for authors with skills and expertise related to the topics of interest by using the query “diabetes AND (wearable OR mobile).” On the basis of the list of retrieved authors, we identified their studies within the scope of wearable and mobile sensing in diabetes.

**Queries’ results per database.**

**Scopus**

- TITLE-ABS-KEY(diabetes AND ((sensor OR sensing OR accelerometer OR gyroscope OR “proximity sensor” OR “light sensor” OR pedometer OR barometer OR gps OR camera OR “humidity sensor” OR magnetometer OR compass OR microphone OR mic OR nfc OR Bluetooth OR Wi-Fi OR fingerprint OR sms OR “phone call” OR “call log”) AND ((wearable OR “smart watch” OR smartwatch OR “fitness band” OR “flexible band” OR wristband OR “smart insole” OR bracelet) OR (mobile OR smartphone OR “smart phone” OR cellphone OR “cell phone” OR mobilephone OR “mobile phone”))))

**Web of Science**

- (ts = (diabetes AND ((sensor$ OR sensing OR accelerometer$ OR gyroscope$ OR “proximity sensor$” OR “light sensor$” OR pedometer$ OR barometer$ OR gps OR camera$ OR “humidity sensor$” OR magnetometer$ OR compass OR microphone$ OR mic OR nfc OR Bluetooth OR Wi-Fi OR fingerprint OR sms OR “phone call$” OR “phone$ call” OR “call log$”) AND ( (wearable$ OR “smart watch*” OR smartwatch* OR “fitness band$” OR “flexible band$” OR wristband$ OR “smart insole$” OR bracelet$) OR (mobile$ OR smartphone$ OR “smart phone$” OR cellphone$ OR “cell phone$” OR mobilephone$ OR “mobile phone$”)))))

**PubMed**

- ((diabetes AND ((sensor OR sensing OR accelerometer OR gyroscope OR “proximity sensor” OR “light sensor” OR pedometer OR barometer OR gps OR camera OR “humidity sensor” OR magnetometer OR compass OR microphone OR mic OR nfc OR Bluetooth OR Wi-Fi OR fingerprint OR sms OR “phone call” OR “call log”) AND ((wearable OR “smart watch” OR smartwatch OR “fitness band” OR “flexible band” OR wristband OR “smart insole” OR bracelet) OR (mobile OR smartphone OR “smart phone” OR cellphone OR “cell phone” OR mobilephone OR “mobile phone”))))[TitleAbstract])

### Study Selection

Manuscripts resulting from the database search (WoS, Scopus, and PubMed) and the hand search (ResearchGate) were downloaded and merged, and duplicates were removed. A 2-stage process was applied for the analysis of the manuscripts. In the first stage, 2 of the authors (CR and OB) screened the manuscripts based on the eligibility criteria, using title and abstract. In the second stage, the same authors fully reviewed the manuscripts resulting from the first stage and selected those meeting the eligibility criteria. During both the initial screening and full-text screening for eligibility, the 2 authors processed all the papers independently and discussed their observations before making a definitive decision. In the event of disagreement, a third reviewer (CV) was assigned, and a final decision was made based on the majority vote.

### Eligibility Criteria

Studies were included if related to DM and if data were collected using sensors from wearable devices or smartphones and transmitted wirelessly. Hence, studies that were not related to DM were directly excluded. Those related to DM but where data were not collected using wearables or smartphones or where data were not transmitted wirelessly were also excluded. The inclusion criteria for both disease and technology are explained below.

According to the considered disease, a manuscript was included if it focused exclusively on DM, meaning that the main clinical topic of the study was DM; it was related to DM complications, that is, the main clinical topic of the study was a complication (or several) resulting from DM; it studied DM in combination with another disease, in other words, the main clinical topic of the study was the relation between DM and another condition such as cardiovascular disease; and patients with DM were used as a case study, namely, a clinical solution for multiple conditions was proposed, but the evaluation was performed on patients with DM.

According to the technology, the definition of *wearable* used for the inclusion criteria was “electronic device with micro-controllers, that can be incorporated into clothing or worn on the body as implants or accessories” [31]. These devices can be either commercial, medical, or prototypes. The definition of *smartphone* for the inclusion criteria was the given by the Oxford dictionary: “a mobile phone that performs many of the functions of a computer, typically having a touchscreen interface, internet access, and an operating system capable of running downloaded apps.” Moreover, both wearables and mobile devices must send the monitored data wirelessly to the storage endpoint to meet the inclusion criteria.

Studies meeting the disease and technology inclusion criteria were also excluded if they were oriented to the intervention without an actual monitoring of DM-related parameters; they were technology centered, namely, the solution was not applied to a clinical case study; the proposed solution was not tested; similar studies under a different title were already considered; and the manuscript was not available.

Only English manuscripts in engineering and computer science areas, of article or proceedings type, and published between January 2010 and July 2020 (both inclusive) were included.

### Quality Assessment

The 9-point NOS was used to score the included manuscripts. Nonrandomized studies, including case-control and cohort studies, were independently scored by 2 authors (CR and JRR). Disagreements were discussed and resolved.

## Results

### Overview

**Figure 1:**
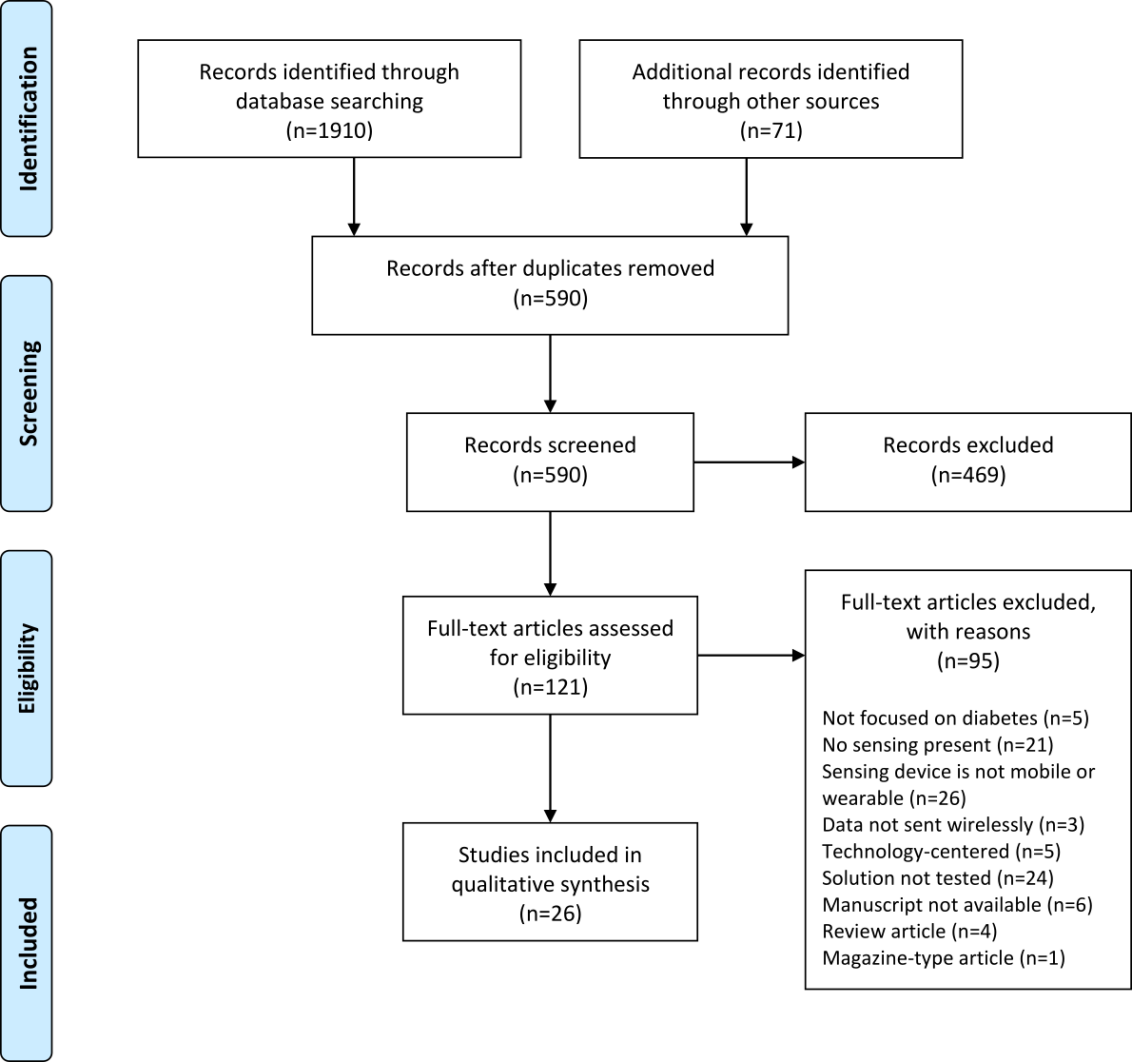
Search and selection of manuscripts using the PRISMA (Preferred Reporting Items for Systematic Reviews and Meta-Analyses) guidelines.

The query used in the Scopus, WoS, and PubMed databases resulted in 960, 627, and 323 references, respectively. A total of 71 manuscripts were identified through other sources. After applying the PRISMA guidelines (Figure 1), 26 publications were eventually included in the full review, 4 exclusive from Scopus [32-35]; 8 from Scopus and WoS [36-43]; 2 from Scopus and PubMed [44,45]; 1 from WoS and PubMed [46]; 9 from Scopus, WoS, and PubMed [47-55]; and 2 available in both Scopus and PubMed and other sources [56,57].

The process used to achieve the 26 publications included in this review is as follows. A total of 590 original studies were obtained after merging the results from the databases and eliminating duplicates. Up to 79.5% (469/590) of the manuscripts were excluded after screening the title and abstract. Of the 469 manuscripts, 243 (51.8%) were not focused on DM, with a majority of papers where the term *diabetes* was part of the subject but most often just used as an exemplary use case; in 55 (11.7%) manuscripts, no sensing took place; in 4 (0.9%) of the manuscripts, the sensing device was not considered mobile or wearable; 97 (20.7%) manuscripts were mainly focused on interventions without an actual monitoring of DM-related parameters; 18 (3.9%) manuscripts were exclusively centered on the technology and did not have a clinical application; 1 (0.2%) study proposed a solution but simply at a conceptual level; 4 (0.9%) studies were found to be very similar to other considered studies despite being entitled differently; 11 (2.4%) were proceedings reviews; and finally 36 (7.7%) manuscripts were reviews or surveys.

The 121 manuscripts resulting from the previous screening were fully analyzed. A total of 78.5% (95/121) of the manuscripts were excluded following the same criteria mentioned above: 5% (5/95) studies were not focused on DM; in 22% (21/95) of the studies, no sensing was performed; in 27% (26/95) of the studies, the sensing device was not considered mobile or wearable; in 3% (3/95) of the studies, the data were not sent wirelessly; 5% (5/95) of the studies were centered on technology; 25% (24/95) of the studies were not properly tested, that is, some studies did not show enough scientific maturity in their tests and others did not involve patients with DM; 6% (6/95) of the manuscripts were not available; 4% (4/95) of the manuscripts were reviews or surveys; and finally, 1% (1/95) of the manuscript was a magazine. As a result, 21.5% (26/121) of the manuscripts were selected to be reviewed in this study.

Of the 26 selected studies, 22 (85%) studies were assessed in terms of quality (Multimedia Appendix 1 [32-34,36-38,40-43,45-54,56,57]). A total of 19 (73%) studies were deemed as cohort studies [33,34,36-38,40-42,45-54,57]. Moreover, 12% (3/26) of studies were identified as case-control studies [32,43,56]. The remaining 15% (4/26) of studies were not identified as nonrandomized [35,39,44,55]; therefore, they were not included in the quality assessment. Of the assessed studies, 41% (9/22) were rated 1 star [36,37,40,43,46-49,51], 55% (12/22) were rated 2 stars [32-34,38,41,42,45,50,52-54,56], and only 5% (1/22) study was rated 4 stars [57]. None of the studies obtained any star for the comparability criteria. The ratings were generally low, which is nevertheless explained by the fact that the selected studies were not of an intervention type.

Some general statistics and quality indicators were obtained from the selected studies. Overall, 62% (16/26) manuscripts were published in journals, whereas (10/26) manuscripts were included in conference proceedings. Moreover, 23% (6/26) of the articles were published in journals ranked in quartile 1, 4% (1/26) in quartile 2, 8% (2/26) in quartile 3, and 4% (1/26) in quartile 4% (1/26) according to the Journal Citation Reports (WoS). In addition, 19% (5/26) of the articles were published in journals ranked in quartile 1, 23% (6/26) in quartile 2, 4% (1/26) in quartile 3, and 4% (1/26) in quartile 4 according to the SCImago Journal Rank (Scopus). It was observed that cross-national research teams were present on many occasions. These teams had a European member 14 times, had a member from the Unites States 11 times, and had an Asian member 8 times. The majority of the studies (21/26, 81%) were published between 2017 and 2019, as shown in Multimedia Appendix 2.

Close to half of the manuscripts (12/26, 46%) did not focus on a specific type of DM, almost a third of the studies (8/26, 31%) were related to T1D, 5 were associated with T2D (5/26, 19%), and 1 study dealt with GD (1/26, 4%). The majority of the studies (25/26, 96%) were some type of trial or longitudinal study, with sample sizes ranging from 1 to 100 participants with an approximate duration of up to 140 days. Patients with DM were involved in approximately two-third of these trials (17/26, 65%). More than half of the studies used only wearables to carry out their objectives (15/26, 58%), approximately one-fifth of the studies used only smartphones (5/26, 19%), and 6 studies used both wearables and smartphones in combination (6/26, 23%). The most common sensor used in the studies was the ACC (19/26, 73%).

In the following sections, we report the findings extracted from the analysis of all the reviewed studies. The dimensions for characterization are medical classification, DM type, research goals, devices, sensors, data processing, *privacy and security*, and *study’s characteristics*. *Medical classification* refers to studies that focused exclusively on DM, related to DM complications, studied DM in combination with another disease, or used patients with DM as a case study. *DM type* can be T1D, T2D, GD, or not specified. *Research goals* refer to activity recognition, diagnosis or prediction of the onset or evolution of DM, finding associations among DM-related variables, or simply measuring DM-related parameters. *Devices* can be *wearable*, *smartphone*, *wearable and smartphone*. *Sensors* refer to ACC, glucose monitor (GM), HR monitor, etc. *Data processing* is categorized into statistical analysis, machine learning (ML), ontologies, or none. *Privacy and security* refer to mechanisms used to preserve users’ anonymity, ethical aspects, and data protection. *Study’s characteristics* refer to size, length, and subjects’ characteristics.

### Medical Classification, DM Type, and Research Goals

More than half of the selected studies, namely, 62% (16/26) of manuscripts were exclusively focused on DM [32,34-40,45-47,50,51,53-55]. Other studies addressed some complications that DM can lead to, specifically, diabetic foot ulcer (DFU) in 15% (4/26) of articles [33,48,56,57] and diabetic peripheral neuropathy (DPN) in 4% (1/26) of studies [56]. Three additional studies (12%) investigated DM in conjunction with other diseases that were not a complication of DM itself but were closely related to it. Nguyen et al [41] considered cardiovascular diseases besides DM, whereas in the study by Sarda et al [52], the association between DM and depression was explored. Sevil et al [42] found an association between acute psychological stress and glucose dynamics in patients with T1D. Some of the screened manuscripts were not focused on a specific disease, but their results can be applied to different health domains, such as obesity, nutrition, hypertension, and DM. Furthermore, 12% (3/26) of these studies were included in the final set of selected articles because they used patients with DM as a case study [43,44,49].

According to the DM type, 4% (1/26) of studies were related to GD [49], 31% (8/26) of studies were related to T1D [32,38,42,45,50,51,53,54], 19% (5/26) of studies were related to T2D [34,39,43,46,55], and 46% (12/26) of studies did not mention any specific type of DM [33,35-37,40,41,44,47,48,52,56,57].

As for the research goals of the selected studies, 15% (4/26) of manuscripts aimed to detect physical activity patterns relevant to patients with DM, such as walking, running, or sleeping [36,37,41,47]. Three studies [36,37,47] focused on recognizing eating, exercising, and sleeping activities by using body ACC and HR collected via a smartphone and a chest strap. In addition, 2 studies [37,47] collected sound, location, velocity, and respiration rate (RR) for a similar purpose. Nguyen et al [41] also used a chest band and ambient sensors to measure ACC, continuous GL, body temperature, room temperature, or humidity data, which are used for fall detection and remote monitoring of DM-related parameters.

Overall, 23% (6/26) of manuscripts [34,35,38,39,48,53] focused on diagnosing or predicting affections related to DM from sensor data. Calbimonte et al [38] predicted glycemic events by collecting ECG, RR, and ACC from a chest strap. Fraiwan et al [48] created a mobile thermal imaging system to indicate the potential development of a DFU. Reddy et al [39] aimed to create a classification system to identify an individual’s diabetic status (healthy or diabetic) using photoplethysmogram data collected from both a smartphone and pulse oximeter. Ramazi et al [34] predicted the progression of T2D by collecting the physical activity and GL from a wristband and a CGM. Garcia et al [35] diagnosed DM from facial images captured using a smartphone. Finally, Rodriguez-Rodriguez et al [53] predicted GL using the CGM data alone.

A total of 38% (10/26) of studies were aimed at finding an association between variables or diseases related to DM [32,42,44,45,50-52,54,56,57]. Najafi et al [44] found some links among biometric variables, postural and balance control, from the quaternion data collected via a hip-worn strap prototype. Turksoy et al [50] determined which variable was the most useful for inclusion in a future artificial pancreas using data such as ACC, GL, and HR from various wearable devices such as an armband, a CGM, and a smartwatch. Faccioli et al [45] studied the relation between physiological information on physical exercise and glucose models, mainly from ACC and GL data sensed via an activity tracker and CGM. Similarly, Merickel et al [32] found a relation between the driver state and the health and physiological state of patients with T1D using a wristwatch and a CGM to obtain primarily ACC, GL, and HR. Likewise, Sarda et al [52] analyzed the relation between some variables sensed by a smartphone, such as mobility, sleep patterns, and location, and the symptoms of depression with DM. Sevil et al [42] found an association between acute psychological stress and glucose dynamics. Similarly, Sanz et al [54] sought to find associations between different signals provided by 3 different wearable devices and the accuracy of a CGM device during aerobic exercise. Two studies found associations between diseases. On the one hand, Grewal et al [56] found a relation between DPN, with no other complications, and DPN combined with DFU using spatiotemporal parameters from a lower limb band. On the other hand, Razjouyan et al [57] found a relation between physiological stress and DFU healing speed via the monitoring of ACC, HR, and RR from a chest strap. Finally, Groat et al [51] attempted to correlate biometric and physiological variables sensed by a CGM and a wristband through manual entries on a mobile app.

Finally, 23% (6/26) of manuscripts had the principal goal of simply measuring data relevant to DM [33,40,43,46,49,55]. McLean et al [49] sensed physical proximity, physical activity, sedentary behavior, location, and orientation from a smartphone. Bartolic et al [40] primarily used a wristband and a smartphone to monitor daily activity, sleep duration, and calorie consumption, among others. McMillan et al [46] collected physical activity data, sedentary behavior, and continuous GL using a thigh-worn wearable and a flash glucose monitor (FGM). Rescio et al [33] sensed the temperature and pressure of the plantar foot with a smart insole. Zherebtsov et al [43] quantified changes in the microcirculatory blood flow in tissues using a wristband. Whelan et al [55] measured the usage, feasibility, and acceptability of behavioral and physiological self-monitoring technologies with 2 different wristbands and an FGM.

All details about medical classification, DM type, and research goals of the reviewed manuscripts are provided in Table 1.

**Table 1:** Summary of medical topics, diabetes types, and research goals, ordered by the year of publication.

Manuscript	Medical classification	Diabetes type	Research goals
Najafi et al (2010) [44]	Patients with DM^a^ used as case study	Not specified	Find association between posture and balance control among patients with different DM complications
Grewal et al (2013) [56]	DM complication: DPN^b^ and DPN with diabetic foot	Not specified	Find association between DPN and DFU^c^ for gait
Luštrek et al (2014) [36]	Focused on DM	Not specified	Activity recognition of walking, running, cycling, lying, sitting, and standing
Luštrek et al (2015) [37]	Focused on DM	Not specified	Activity recognition of sleeping, home chores, home leisure, eating, and exercising
Cvetković et al (2016) [47]	Focused on DM	Not specified	Activity recognition of working, eating, exercising, and home activities
Calbimonte et al (2017) [38]	Focused on DM	T1D^d^	Predict glycemic events
Fraiwan et al (2017) [48]	DM complication: diabetic foot	Not specified	Diagnose development of DFU
McLean et al (2017) [49]	Patients with DM used as case study	GD^e^	Measure physical proximity, physical activity, and magnetic field strength
Razjouyan et al (2017) [57]	DM complication: diabetic foot	Not specified	Find association between physiological stress response and healing speed among outpatients with active DFU
Reddy et al (2017) [39]	Focused on DM	T2D^f^	Diagnose individual’s diabetic status
Turksoy et al (2017) [50]	Focused on DM	T1D	Find association between biometric variables and changes in glucose concentration
Bartolic et al (2018) [40]	Focused on DM	Not specified	Measure GL^g^, insulin dosage, physical activity, daily movement, and sleep duration and quality
Faccioli et al (2018) [45]	Focused on DM	T1D	Find association between glucose prediction models’ performance
Groat et al (2018) [51]	Focused on DM	T1D	Find association between exercise behavior data with the rate of change in GL
McMillan et al (2018) [46]	Focused on DM	T2D	Measure combined GL data, physical activity, and sedentary behavior
Merickel et al (2018) [32]	Focused on DM	T1D	Find association between pattern of glucose and at-risk pattern of vehicle acceleration behavior
Nguyen Gia et al (2019) [41]	DM in conjunction with other diseases: DM+cardiovascular disease	Not specified	Activity recognition of fall detection and remote health monitoring
Rescio et al (2019) [33]	DM complication: diabetic foot	Not specified	Measure temperature and pressure of the plantar foot
Sarda et al (2019) [52]	DM in conjunction with other diseases: DM+depression	Not specified	Find association between smartphone-sensing parameters and symptoms of depression
Ramazi et al (2019) [34]	Focused on DM	T2D	Predict the progression of T2D
Garcia et al (2019) [35]	Focused on DM	Not specified	Diagnose DM from facial images
Sevil et al (2019) [42]	DM in conjunction with other diseases: DM+acute psychological stress	T1D	Find the association between acute psychological stress and the glucose dynamics
Zherebtsov et al (2019) [43]	Patients with DM used as case study	T2D	Measure the changes in the microcirculatory blood flow of healthy patients and patients with T2D
Rodriguez-Rodriguez et al (2019) [53]	Focused on DM	T1D	Predict blood GL for T1D with limited computational and storage capabilities using only CGM^h^ data
Sanz et al (2019) [54]	Focused on DM	T1D	Find the association between different signals provided by 3 different wearables devices and the accuracy of a CGM device during aerobic exercises
Whelan et al (2019) [55]	Focused on DM	T2D	Measure the use, feasibility, and acceptability of behavioral and physiological self-monitoring technologies in individuals at risk of developing T2D

^a^DM: diabetes mellitus.

^b^DPN: diabetic peripheral neuropathy.

^c^DFU: diabetic foot ulcer.

^d^T1D: type 1 diabetes.

^e^GD: gestational diabetes.

^f^T2D: type 2 diabetes.

^g^GL: glucose level.

^h^CGM: continuous glucose monitor.

### Devices and Sensors

A total of 58% (15/26) of studies used wearable devices for monitoring tasks [32-34,38,41-44,46,50,53-57], 3 of which were research prototypes, namely, a hip-worn strap [44], a chest strap [41], and a smart insole [33]. Of the remaining 12 studies, 5 (42%) used wearable of a commercial type; those were chest straps (Bioharness 3, Zephyr) [38,57], a lower limb band (LEGSys, BioSensics LLC) [56], a wristband (AMT-LAZMA 1, Aston Medical Technology Ltd) [43], and an FGM (FreeStyle Libre, Abbott Diabetes Care) [53]. In 23% (6/26) of studies, the authors used various commercial wearable devices in combination: an armband (SenseWear, BodyMedia), 2 CGMs (Guardian Real-Time, Medtronic, and Dexcom G4 Platinum, Dexcom, Inc), a chest strap (Bioharness 3, Zephyr), and a smartwatch (Mio Alpha, MIO Global) [50]; a thigh-worn (activPAL, PAL Technologies Ltd) and a FGM (FreeStyle Libre, Abbott Diabetes Care) [46]; a wristwatch (model not specified) and a CGM (Dexcom G4 Platinum, Dexcom, Inc) [32]; a wristband (ActiGraph, model not specified, ActiGraph LLC) and a CGM (Dexcom G4 Platinum, Dexcom, Inc) [34]; a wristband (Empatica E4, Empatica Inc) and a CGM (Dexcom G5, Dexcom, Inc) [42]; 3 wristbands (Fitbit Charge HR, Fitbit, Inc; Microsoft Band 2, Microsoft Corporation; and Polar RCX3, Polar Electro) and a CGM (Enlite-2, Medtronic Minimed) [54]; and 2 wristbands (Fitbit Charge 2, Fitbit, Inc, and ActiGraph wGT3x-BT, ActiGraph LLC) and a FGM (FreeStyle Libre, Abbott Diabetes Care) [55].

Smartphones were used in 19% (5/26) of studies for sensing purposes: a Samsung Galaxy S6 Edge Plus (Samsung) [48], a Nexus 5 (LG Corporation & Google, LLC) [39], an iPhone 7 (Apple Inc) [35], and no specific details were found for the other 2 studies [49,52]. A total of 6 studies used both wearables and smartphones to acquire data [36,37,40,45,47,51]. In 3 studies [36,37,47], a smartphone (model not specified) and a chest strap (model not specified) were used. Bartolic et al [40] used a smartphone (model not specified) with a wristband (Fitbit fitness bracelet, Fitbit, Inc). Faccioli et al [45] used a smartphone (model not specified), an activity tracker (model not specified), and a CGM (model not specified). Groat et al [51] used a smartphone (model not specified), a wristband (Fitbit Charge HR, Fitbit, Inc), and a CGM (Enlite, Medtronic) to perform the monitoring task.

A total of 30 different types of sensors were used to acquire data from the reviewed studies. The ACC stands out as the sensor most widely used across studies, namely, in 73% (19/26) of studies [32,34,36-38,40-42,44-47,49,50,52,54-57]. This sensor has been primarily used for the automatic recognition of activities such as motion, walking, running, exercise, cycling, standing, sitting, sleep, step count, and fall detection. The second most used sensor is the GM, which was used to monitor glucose in 46% (12/26) of the most recently published studies [32,34,40-42,45,46,50,51,53-55]. The subject’s glucose was measured using different types of GM sensors: continuously with a CGM [32,34,41,42,45,50,51,54], with an FGM [46,53,55], and manually with a Bluetooth glucometer [40] and with a glucose and L-lactate analyzer [54]. Moreover, HR monitor was included in 27% (7/26) studies [32,36,40,50,51,54,55] to measure the HR of the subjects and infer mainly exercise intensity, calorie consumption, and activity recognition. Some apps can also be considered as sensors when used to encode data manually. For example, Groat et al [51] used an app to be operated by the subjects involved in the trials to self-track exercise behavior and rate of change in GLs.

All details about the devices and sensors used in the reviewed manuscripts are provided in Table 2. A summary of the sensor use for each study is available in Multimedia Appendix 3 [32-57].

**Table 2:** Summary of devices used, ordered by the year of publication.

Studies and devices	Devices, sensors, measurement	Purpose
Najafi et al (2010) [44]	Wearable: hip-worn strap (*prototype*^a^) Triaxial ACC^b^ (quaternions) Triaxial gyroscope (quaternions) Triaxial magnetometer (quaternions) Complementary: pressure platform (*Emed-x system, Novel Inc*) Pressure sensor (area of sway)	Recognize the motion of ankle and hip joints in 3 dimensions
Grewal et al (2013) [56]	Wearable: lower limb band (*LEGSys, BioSensics LLC*) ACC (acceleration) Gyroscope (angular velocity)	Gait detection
Luštrek et al (2014) [36]	Smartphone (*model not specified*) ACC (acceleration) Wearable: chest strap (*model not specified*) ACC (acceleration) HRM^c^ (HR^d^)	Smartphone location detection; activity recognition: walking, running, cycling, lying, sitting, and standing; and energy expenditure estimation
Luštrek et al (2015) [37]	Smartphone (*model not specified*) ACC (acceleration, location, HR, and RR^e^) Microphone (sound) GPS (location and velocity) Wi-Fi (location) Wearable: chest strap (*model not specified*) ACC (acceleration) ECG^f^ (HR and RR)	Activity recognition: sleep, exercise, work, transport, eating, home, and outdoor
Cvetković et al (2016) [47]	Smartphone (*model not specified*) ACC (acceleration) Microphone (sound) GPS (location and velocity) Wi-Fi (location) Wearable: chest strap (*model not specified*) ACC (acceleration) ECG (HR and RR)	Activity recognition: sleep, exercise, work, transport, eating, home, and out
Calbimonte et al (2017) [38]	Wearable: chest strap (*Bioharness 3, Zephyr*) ACC (acceleration) ECG (HB^g^ fiducial points location, ST^h^ segment shape, QTc^i^ interval, HR, and RR)	Generate 2 semantic models: physiological and energy expenditure for classifying hypoglycemic events
Fraiwan et al (2017) [48]	Smartphone (*Samsung Galaxy S6 Edge Plus, Samsung*) Complementary: infrared thermal camera (*FLIR ONE, FLIR Systems, Inc*) Infrared sensor (thermal images) Camera (standard image)	Recognize change of temperature on the feet
McLean et al (2017) [49]	Smartphone (*model not specified*) ACCs (acceleration) GPS (location) Wi-Fi (location) Camera (photo) Magnetometer (magnetic field strength) Bluetooth (physical proximity)	Quantify physical proximity, sedentary behavior, vehicle use, and location
Razjouyan et al (2017) [57]	Wearable: chest strap (*Bioharness 3, Zephyr*) ACC (acceleration) ECG (HR, RR, and core body temperature)	Detection of physiological stress of the patient
Reddy et al (2017) [39]	Smartphone (*Nexus 5, LG Corporation and Google, LLC*) Camera and flash (PPG^j^) Complementary: peripheral pulse oximeter (*model not specified*) Pulse oximeter (PPG)	Discriminate between diabetic and healthy individuals
Turksoy et al (2017) [50]	Wearable: armband (*SenseWear, BodyMedia*) ACC (acceleration) Thermometer (skin temperature and near-body temperature) Galvanometer (galvanic skin response) Heat flux (rate of heat dissipating from the body) Wearable: CGM (*Guardian Real-Time, Medtronic*) GM^k^ (GL^l^) Wearable: CGM (Dexcom *G4 Platinum, Dexcom, Inc*) GM (GL) Wearable: chest strap (*Bioharness 3, Zephyr*) ACC (acceleration) ECG (HR) Wearable: smartwatch (*Mio Alpha, MIO Global*) HRM (HR) Complementary: open-circuit spirometry metabolic cart system (*True One, Parvo Medics*) Expired gases (O2 and CO2)	Find a correlation between biometric changes and glucose concentrations during exercise
Bartolic et al (2018) [40]	Smartphone (*model not specified*) App (insulin doses) Wearable: wristband (*Fitbit fitness bracelet, Fitbit, Inc*) ACC (acceleration) HRM (HR) Complementary: glucometer (*Contour Next One, Ascensia Diabetes Care*) GM (GL)	Quantify physical activity, daily movement, sleep duration and quality, calorie consumption, insulin dosages, and continuous GL
Faccioli et al (2018) [45]	Smartphone (*model not specified*) App (carbohydrates count) Wearable: activity tracker (*model not specified*) ACC (acceleration) Wearable: CGM (*model not specified*) GM (continuous GL)	Quantify step count, continuous GL, and carbohydrate intake
Groat et al (2018) [51]	Smartphone (*model not specified*) App (exercise behavior) Wearable: wristband (*Fitbit Charge HR, Fitbit, Inc*) HRM (HR) Wearable: CGM (*Enlite, Medtronic*) GM (continuous GL)	Quantify exercise behavior measured via a wristband and an app to compare with the rate of change in GL recorded by a CGM
McMillan et al (2018) [46]	Wearable: thigh-worn (*activPAL, PAL Technologies Ltd)* ACC (acceleration) Inclinometer (acceleration) Wearable: FGM (*FreeStyle Libre, Abbott Diabetes Care*) GM (continuous GL)	Quantify step count, cadence and postural transitions and energy expenditure estimates, and continuous GL
Merickel et al (2018) [32]	Wearable: wristwatch (*model not specified*) ACC (acceleration) HRM (HR) Wearable: CGM (Dexcom *G4 Platinum, Dexcom, Inc*) GM (continuous GL) Complementary: vehicle sensor instrumentation packages (*model not specified*) Camera (video) GPS (vehicle acceleration and speed) OBD^m^ sensor (vehicle acceleration and speed)	Compare the driving behavior from drivers with and without T1D^n^
Nguyen Gia et al (2019) [41]	Wearable: chest strap (*prototype*) ACC (acceleration) Gyroscope (angular velocity) Magnetometer (magnetic field) ECG (QT^o^ intervals and HR) GM (continuous GL) Thermometer (body temperature) Complementary: ambient sensors (*prototype*) Ambient sensor (room temperature, humidity, and air quality)	Monitor DM^p^ and ECG, and report abnormalities: fall, very low or high GL, and abnormal HR in real time without interfering with the patient’s daily activities
Rescio et al (2019) [33]	Wearable: smart insole (*prototype*) Infrared thermometer (plantar temperature) Pressure sensor (pressure)	Monitor temperature and pressure of the plantar foot
Sarda et al (2019) [52]	Smartphone (*model not specified*) ACC (acceleration) Call logs (communication) GPS (location) Ambient light sensor (ambient light)	Activity recognition: mobility, sleep, and social interaction
Ramazi et al (2019) [34]	Wearable: CGM (*Dexcom* *G4 Platinum, Dexcom, Inc*) GM (continuous GL) Wearable: wristband (*model not specified; ActiGraph, ActiGraph LLC*) ACC (acceleration)	Quantify GL, traveled steps, and physical activity: sitting, standing, and lying
Garcia et al (2019) [35]	Smartphone (*iPhone 7, Apple Inc*) Camera (standard image)	Capture facial images
Sevil et al (2019) [42]	Wearable: wristband (*Empatica E4, Empatica Inc*) ACC (acceleration) PPG (blood volume pulse) Galvanometer (galvanic skin response) Infrared thermopile (skin temperature) Wearable: CGM (*Dexcom G5, Dexcom, Inc*) GM (continuous GL)	Estimate acute psychological stress effect index and GL
Zherebtsov et al (2019) [43]	Wearable: wristband (*AMT-LAZMA 1, Aston Medical Technology Ltd*) Laser Doppler flowmetry (Doppler shift)	Quantify changes in the microcirculatory blood flow in tissues
Rodriguez-Rodriguez et al (2019) [53]	Wearable: FGM (*FreeStyle Libre, Abbott Diabetes Care*) GM (continuous GL)	Quantify GL for the creation of a database for further processing by the prediction models
Sanz et al (2019) [54]	Wearable: wristband (*Fitbit Charge HR, Fitbit, Inc*) ACC (acceleration) HRM (HR) Altimeter (altitude) Wearable: wristband (*Microsoft Band 2, Microsoft Corporation*) HRM (HR) Skin temperature (skin temperature) Galvanometer (galvanic skin response) ACC (acceleration) Wearable: wristband (*Polar RCX3, Polar Electro*) HRM (HR) Wearable: CGM (*Enlite-2, Medtronic Minimed*) GM (continuous GL) Complementary: glucose and L-lactate analyzer (*YSI 2300 Stat Plus Glucose Analyzer, YSI Incorporated Life Sciences*) GM (GL)	Quantify number of steps walked, number of floors of stairs climbed, exercise intensity, calories burned, and skin electrodermal activity
Whelan et al (2019) [55]	Wearable: wristband (*Fitbit Charge 2, Fitbit, Inc*) ACC (acceleration) Altimeter (altitude) HRM (HR) Wearable: wristband (*ActiGraph wGT3x-BT, ActiGraph LLC*) ACC (acceleration) Wearable: FGM (*FreeStyle Libre, Abbott Diabetes Care*) GM (continuous GL)	Quantify number of steps walked, distance traveled, HR, calories expended, flights of stairs climbed, and GL

^a^Text in italic represents model and company of each devices in that order. In the cases of no specification on the correspondent manuscript “Model not specified” it is stated.

^b^ACC: accelerometer.

^c^HRM: heart rate monitor.

^d^HR: heart rate.

^e^RR: respiration rate.

^f^ECG: electrocardiogram.

^g^HB: heartbeat.

^h^ST: electrocardiogram measurement ST interval.

^i^QTc: corrected electrocardiogram measurement QT interval.

^j^PPG: photoplethysmogram.

^k^GM: glucose monitor.

^l^GL: glucose level.

^m^OBD: on-board diagnostics device.

^n^T1D: type 1 diabetes.

^o^QT: electrocardiogram measurement QT interval.

^p^DM: diabetes mellitus.

### Data Processing

Except for 12% (3/26) of studies [33,46,49], most manuscripts included some type of processing to the collected data. In 42% (11/26) of studies, the processing mainly consisted of statistical analysis [32,40,43-45,50,51,54-57]; in 23% (6/26) of studies, the authors used ML techniques [35-37,39,41,47], and in 15% (4/26) of studies [34,42,52,53], both statistical analysis and ML were used. Fraiwan et al [48] used image processing to detect areas with varying body temperature, and Calbimonte et al [38] used ontologies for diagnosing glycemic events.

The studies without specific data processing were rather oriented to simply collect data. ML was primarily used in the reviewed investigations for activity recognition and diagnosis tasks. Statistical analyses were predominantly aimed at finding associations between behavioral and physiological variables with DM conditions.

Some authors created their own algorithm to attain the study objective. Cvetković et al [47] developed 4 new methods to recognize human activity: person-dependent, person-independent, person-independent with person-specific data, and person-independent and person-specific models combined with heuristics and multiclassifier adaptive training. Merickel et al [32] developed their own procedure to eliminate the spurious values of GLs and to discretize them. Nguyen et al [41] created new algorithms for HR and the QT interval extraction from the ECG for activity status categorization and for fall detection. Finally, Ramazi et al [34] developed a new algorithm for different sensor signal synchronization.

Some studies used additional data, not collected passively from mobile or wearable devices, to achieve their goal. One example is the study by Razjouyan et al [57], where the authors collected clinical information such as depression scale, numeric pain scale, and glycated hemoglobin level by using questionnaires, in addition to demographic information. Similarly, Ramazi et al [34] used clinical information, such as triglycerides, low-density lipoprotein cholesterol, high-density lipoprotein cholesterol, and very low-density lipoprotein cholesterol, and demographic information to improve the prediction of T2D progression in patients. Furthermore, in the study by Turksoy et al [50], the diet and physical activity of the subjects were documented manually throughout the remainder of the study period. Sarda et al [52] collected sociodemographic information, such as gender, marital status, occupation, or education, to perform descriptive analysis to understand the societal representation of the participants. Finally, Sevil et al [42] recorded, besides sensor data, nutrition data (carbohydrate ingestion times, content, and amount); demographic information; and other information, such as mood, sleep information, and feelings of anxiety or depression, using questionnaires.

All details about data processing for the reviewed manuscripts are provided in Table 3.

**Table 3:** Summary of processing techniques, privacy, and security ordered by the year of publication.

Manuscript	Statistical methods	Machine learning methods	Privacy	Security
Najafi et al (2010) [44]	Pearson correlation coefficient, paired *t* test, and intraclass correlation coefficient	None	Study approved by the local ethics committee	Not described
Grewal et al (2013) [56]	Statistical fluctuation, SD, and coefficient of variation	None	Study approved by the local ethics committee. All participants signed an informed consent form before participating in the study	Not described
Luštrek et al (2014) [36]	None	RF^a^ and support vector regression algorithm	Not described	Not described
Luštrek et al (2015) [37]	None	Spectral centroid, zerocrossing, mel frequency cepstral coefficient, linear predictive coding, and method of moments values of the sound signals; clustering of Wi-Fi and GPS data; new algorithm of acceleration data; naive bayes, logistic regression, SVM^b^, RF, RIPPER^c^, adaboost, and bagging for activity recognition tasks; and event calculus for interpreting recognized activities	Sound from the smartphone microphone is recorded in fractions of 100 ms per second	Not described
Cvetković et al (2016) [47]	None	Spectral centroid, zerocrossing, mel frequency cepstral coefficient, linear predictive coding, and method of moments values of the sound signals; clustering of Wi-Fi and GPS data; new algorithm of acceleration data; 5 new algorithms for activity recognition task; and symbolic rules to refine confused predictions	Sound from the smartphone microphone is recorded in fractions of 100 ms per second	Not described
Calbimonte et al (2017) [38]	None	Normalized least mean squares, ontology, and RDF^d^ stream processing engine (CQELS^e^ continuous evaluation)	Not described	Not described
Fraiwan et al (2017) [48]	None	Otsu thresholding technique and point-to-point mean difference technique	Authors referred to “Ethics approval and consent to participate” as “Not applicable”	Not described
McLean et al (2017) [49]	None	None	Not described	Data are stored on the phone and uploaded in an encrypted form
Razjouyan et al (2017) [57]	Analysis of variance, root mean square of successive R-wave to R-wave intervals, power spectrum density of time series representing R-wave to-R-wave intervals, receiver operating characteristic, and area under the curve	None	Not described	Not described
Reddy et al (2017) [39]	None	SVM, artificial neural network, and classification and regression trees	Not described	Not described
Turksoy et al (2017) [50]	Partial least squares, regression, and variable importance in projection	None	Not described	Not described
Bartolic et al (2018) [40]	Trading view and minimum and maximum values	None	Not described	Not described
Faccioli et al (2018) [45]	Black-box linear model, prediction error method, coefficient of determination, and RMSE^f^	None	The trial study and all experimental procedures were approved by the institution’s ethical review board	Not described
Groat et al (2018) [51]	Cohen κ	None	Study approved by the local institutional review board	Not described
McMillan et al (2018) [46]	None	None	Not described	Not described
Merickel et al (2018) [32]	Their own procedures and β regression model	None	All subjects gave informed consent to study participation according to the University of Nebraska Medical Center’s institutional review board’s protocols	Not described
Nguyen Gia et al (2019) [41]	None	Heart rate and the QT^g^ interval extraction, activity status categorization, and fall detection	Not described	Lightweight cryptography
Rescio et al (2019) [33]	None	None	Not described	Not described
Sarda et al (2019) [52]	Descriptive analysis and univariate analysis	SVM, RF, adaboost, extreme gradient boosting, and cross-validation	Not described	All transmissions were in an encrypted form using the HTTPS^h^ secure sockets layer protocol
Ramazi et al (2019) [34]	RMSE	New algorithm for different sensor signal synchronization and long short-term memory deep neural network	Study approved by the local institutional review board	Not described
Garcia et al (2019) [35]	None	KNN^i^ and SVM	Not described	Not described
Sevil et al (2019) [42]	Mean, SD, kurtosis, and mean absolute error	SVM, KNN, linear discriminant, decision tree, and logistic regression	Study approved by the local institutional review board	Not described
Zherebtsov et al (2019) [43]	Statistical significance	None	Study approved by the local institutional review board. Each volunteer gave a voluntary informed written consent to participate in the experiment	Not described
Rodriguez-Rodriguez et al (2019) [53]	RMSE	Autoregressive integrated moving average, RF, and SVM	Study conducted in accordance with the Helsinki Declaration. Study approved by the local ethics committee. Data storage complied with the stricter data protection rules for protecting personal information. All participants were fully informed about the purpose of the experiment and provided written informed consent and assent according to the national regulations	Not described
Sanz et al (2019) [54]	Median, linear regression, and cross-validation	None	Study approved by the local ethics committee	Not described
Whelan et al (2019) [55]	Mean, SD, and frequency	None	All participants provided written informed consent. Study approved by the local ethics advisory committee	Not described

^a^RF: random forest.

^b^SVM: support vector machine.

^c^RIPPER: repeated incremental pruning to produce error reduction.

^d^RDF: resource description framework.

^e^CQELS: continuous query evaluation over linked stream.

^f^RMSE: root mean square error.

^g^QT: electrocardiogram measurement QT interval.

^h^HTTPS: Hypertext Transfer Protocol Secure.

^i^KNN: k-nearest neighbors.

### Privacy and Security

A total of 62% (16/26) of studies addressed privacy or security issues [32,34,37,41-45,47,49,51-56]. Of the 13 studies dealt with privacy aspects to some extent: 10 (77%) studies were approved by a local ethics committee or review boards [34,42-45,51,53-56]; in the study by Merickel et al [32], the authors included informed consent for participants; and in 2 (15%) studies [37,47], the sound from the smartphone microphone was downsampled to 100 ms out of every second to preserve the user’s privacy. Finally, the study by Fraiwan et al [48] was not included in the above 16 studies because it was mentioned that the intervention of an ethics committee was not applicable.

Overall, 19% (3/16) of studies considered security aspects. McLean et al [49] stored the data on the phone and then uploaded the data to a server in an encrypted manner. Nguyen et al [41] used lightweight cryptography to deal with security in the mobile monitoring system, and Sarda et al [52] stated that all transmissions were in an encrypted form using the Hypertext Transfer Protocol Secure (HTTPS) secure sockets layer protocol.

The remaining 35% (19/26) of studies did not mention anything about privacy or security [33,35,36,38-40,46,50,57].

All details about *privacy and security* of the reviewed manuscripts are provided in Table 3.

### Study’s Characteristics

The number of participants involved differed significantly among the studies. The average number of participants was 29 (SD 28.2), calculated from 88% (23/26) of studies that indicated the number of participants [32-37,39,41-47,49-57]. The minimum sample size was 1 subject [46], and the maximum sample size was 100 subjects [35,39]. Furthermore, 12% (3/26) of papers did not specify this number [32,38,48]. The duration of the test phase was standardized to days, namely, an average of 21.5 (SD 35.1) days of duration as computed from the 58% (15/26) of studies that specified this value [32,34,37,39,42,43,45-47,50-53,55,57]. One day was the minimum duration of the study [42,46], and 140 days was the maximum duration of the study [52]. In 13% (2/15) studies [39,43], the duration remained in the order of minutes for a better understanding, and 42% (11/26) of studies did not specify this value at all [33,35,36,38,40,41,44,48,49,54,56].

As for the health status distribution for the 26 studies, 11 (42%) studies involved patients with DM [34,42,45,46,49-54,57], 5 (19%) included healthy subjects [36,41,47,48,55], and 6 (23%) involved both healthy subjects and patients with DM [32,35,39,43,44,56]. Moreover, 12% (3/26) of studies did not precisely describe the health status of the subjects [33,37,40], and 4% (1/26) of studies used an existing data set to test their solution [38].

A total of 31% (8/26) of studies involved subjects of both genders [35,37,47,50-53,55], and 4% (1/26) of studies only involved a male [46]. The remaining 65% (17/26) of studies did not specify the gender of the participants [32-34,36,38-45,48,49,54,56,57]. With respect to the age of the involved subjects, 65% (17/26) of studies provided this value [32-35,39,41,43,46,47,50-57], resulting in an average age of 44.5 (SD 12.2) years. The other 35% (9/26) of studies did not mention any age distribution [36-38,40,42,44,45,48,49].

All details about the *study’s characteristics* for the reviewed manuscripts are provided in Table 4.

**Table 4:** Summary of study topic ordered by the year of publication.

Manuscript	Sample size	Sample type	Duration
Najafi et al (2010) [44]	38	17 diabetic and 21 healthy; gender undefined; and age undefined	Not described
Grewal et al (2013) [56]	39	31 diabetic and 8 healthy; gender undefined; and aged 56.9 (SD 8.2) years	Not described
Luštrek et al (2014) [36]	10	0 diabetic and 10 healthy; gender undefined; and age undefined	Not described
Luštrek et al (2015) [37]	5	Health status undefined; 1 female and 4 males; and age undefined	14 days
Cvetković et al (2016) [47]	9	0 diabetic and 9 healthy; 1 female and 8 males; and aged 24-36 years	14 days
Calbimonte et al (2017) [38]	Not described	External data set	Not described
Fraiwan et al (2017) [48]	Not described	Healthy; gender undefined; and age undefined	Not described
McLean et al (2017) [49]	22	22 diabetic and 0 healthy; gender undefined; and age undefined	Not described
Razjouyan et al (2017) [57]	25	25 diabetic and 0 healthy; gender undefined; aged 59.3 (SD 8.3) years	21 (SD 4) days
Reddy et al (2017) [39]	100	50 diabetic and 50 healthy; gender undefined; and aged 34 (SD 10) years (diabetic) and 41 (SD 13) years (healthy)	5 mnin
Turksoy et al (2017) [50]	26	26 diabetic and 0 healthy; 14 females and 12 males; and aged 24.2 (SD 5.41) years	6 days
Bartolic et al (2018) [40]	Not described	Health status undefined; gender undefined; and age undefined	Not described
Faccioli et al (2018) [45]	6	6 diabetic and 0 healthy; gender undefined; and age undefined	5 days
Groat et al (2018) [51]	12	12 diabetic and 0 healthy; 8 females and 4 males; and aged 48 (SD 13.4) years	30 days
McMillan et al (2018) [46]	1	1 diabetic and 0 healthy; 0 female and 1 male; and aged 68 years	1 day
Merickel et al (2018) [32]	36	20 diabetic and 16 healthy; gender undefined; and aged 21-59 years	28 days
Nguyen Gia et al (2019) [41]	4	0 diabetic and 4 healthy; gender undefined; aged 30 years	Not described
Rescio et al (2019) [33]	5	Health status undefined; gender undefined; and aged 47.2 (SD 12.3) years	Not described
Sarda et al (2019) [52]	46	46 diabetic and 0 healthy; 17 females and 29 males; and aged 35 (SD 12) years	140 days
Ramazi et al (2019) [34]	50	50 diabetic and 0 healthy; gender undefined; and aged 33-78 years	7 days
Garcia et al (2019) [35]	100	50 diabetic and 50 healthy; 58 females and 42 males; and aged 20-87 years	Not described
Sevil et al (2019) [42]	2	2 diabetic and 0 healthy; gender undefined; and age undefined	1 day
Zherebtsov et al (2019) [43]	55	18 diabetic and 37 healthy; gender undefined; and aged 53.2 (SD 11.4) years (diabetic), 19.6 (SD 0.6) years (16 healthy), and 53.2 (SD 11.4) years (21 healthy)	10 min
Rodriguez-Rodriguez et al (2019) [53]	25	25 diabetic and 0 healthy; 11 females and 14 males; and aged 18-56 years	14 days
Sanz et al (2019) [54]	6	6 diabetic and 0 healthy; gender undefined; and aged 36.7 (SD 8.9) years	Not described
Whelan et al (2019) [55]	45	0 diabetic and 45 healthy; 27 females and 18 males; and aged 56 (SD 9) years	42 days

## Discussion

### Principal Findings

The reviewed studies revealed the potential of mobile and wearable technologies in health areas. These technologies can significantly improve the management of conditions for both patients and clinicians for a variety of diseases. DM is not an exception, and growing attention has been paid to the use of these technologies in the recent years. Obtaining objective and continuous measurements is an important advantage of using this technology for patient monitoring. Data are sensed automatically by electronic sensors when the subject is interacting with the mobile or wearable devices both explicitly and implicitly, such as phone calls or step counts, respectively. These technologies most often enable the seamless collection of data, even when the patient is out of the clinic. This is a relevant feature to overcome the drawbacks of classical clinical trials in which subjects are required to stay in labs or clinics, set specific appointments, commute to the doctor’s office, etc. This technology adds a level of objectivity in the monitoring of patients with DM and people in general with respect to traditional clinical questionnaires, which are more dependent on users’ willingness and capacity to answer correctly. In addition, the patient may not remember everything accurately in between doctor visits and hospitalization times. In view of such limitations, the opportunities for the monitoring of DM-related parameters are unprecedented. Nonetheless, it is clear from this review that mobile and wearable technologies have been scarcely exploited for this purpose.

Several studies have not indicated the type of DM. In such studies, the authors refer to the condition simply as *diabetes* or *diabetes mellitus*. We assume that in those cases, the results of the research are generic and can be applied to any type of diabetic. However, there was generally a lack of clinical specifications, perhaps because the majority of studies were published in technological journals and proceedings and, in part, because of the recency of this new field. In either case, such a level of specification is considered of utmost importance because of the differences among the types of diabetes and their decompensations, risk factors, age of onset, and treatment among several others. Complications of the disease may result in both types of DM, but few studies have focused on this subject. DPN was the only complication found in the reviewed studies and in almost all cases related to diabetic foot disease. Some important complications such as retinopathy; nephropathy; and other types of neuropathies, such as autonomic, focal, and proximal, were not considered in any of the selected studies. Few studies have elaborated on T2D, the type encompassing between 90% and 95% of the cases of DM in the world and the only preventable one.

Clinical trials were quite limited or even nonexistent in many of the reviewed studies. In fact, the majority of analyzed contributions had a predominant technological focus, prioritizing systems’ performance or robustness over the impact or applicability of potential clinical outcomes. This explains the remarkably low scores achieved by most of the selected studies in the NOS quality assessment. Most of the reviewed cohort studies did not have a sufficiently representative cohort. Most often, a distinction between exposed and nonexposed cohorts was not clearly made, or even worse, no description of the derivation of the cohort was provided. This lack of detail was also observed for cases and controls. Comparability was also found to be quite limited, as exposed and nonexposed individuals, if any, were not matched in the design, and confounders were either missing or not adjusted for in the analysis. Although outcomes were assessed in a majority of studies, follow-ups were mostly nonexistent or no information was provided whatsoever. Therefore, one of the major weaknesses detected in the reviewed studies is the limited dedication to the clinical validation of the proposed technical solutions.

Mobile and wearable data can shed new light on behavioral and physiological aspects that are difficult to approach in a continuous and unobtrusive manner via standard clinical tests. However, ignoring clinical data is certainly a big mistake. Therefore, combining passive mobile data with clinical data, such as laboratory test results, drug information, or patient demographics, is key for a holistic understanding of the patient’s current and future health status. Thus, it is recommended to perform more extensive clinical tests and validations involving the collection of new data sets. Existing data sets in this area show important flaws such as noninclusion of patients with DM, noninclusion of complementary clinical data, lack of gender diversity, or age variety. Moreover, public sharing of data sets is also considered essential to facilitate the replicability and reproducibility of the studies. Hence, data transparency and openness are encouraged, as in other similar disciplines.

None of the reviewed studies focused on the prevention of DM. This matter is especially important in the case of T2D, the only type of DM that can be prevented. Therefore, developing studies with outcomes that help to detect the disease in the early stages or even before it occurs can result in great progress. Approaching this subject from a holistic perspective could also be key for making new successful findings. This is closely related to the idea of using different data sources to generate more powerful medical models. Combining demographics, nutrition data, medication data, and passive sensor data among other heterogeneous data types can certainly help to realize more impactful and personalized solutions.

Activity recognition is one of the most important areas from which the monitoring of DM-related parameters could benefit. Thus, the research conducted in this new field may not only leverage the results from previous studies but also help in developing and testing new activity detection models. For example, improvements in the recognition of eating activities are needed to calculate food intake automatically.

Most of the studies had technological test phases, but in some cases, their quality was rather questionable. The description was often incomplete, lacked characteristics of the subjects, and did not mention the duration of the tests in several cases. These seem to be characteristics deemed in studies in the early stages. However, many of these studies stated that improvements would be made regarding this aspect in future research.

The studies analyzed in this review applied a variety of devices and sensors. Some case studies only used smartphones, others used only wearables, and others used a combination of them. This shows the ways in which these devices can be used to improve DM control and its complications. However, there is generally a poor description of the devices used in terms of their brand, model, manufacturer, main features, operating system, etc. This is an especially sensitive barrier for the replication of studies and development of follow-up research. Likewise, on some occasions, the sensors embedded in these devices were not explicitly described. Some studies included complementary devices such as a pressure platform, a glucometer, or an ambient sensor, not necessarily wearables, which helped to obtain more complete data and better characterize the patient’s environment. Most often, all devices were merely used to collect data for creating ML models and to find an association among variables or diseases, but in very few cases, the proposed solution was implemented in a realistic use case with long-lasting clinical applications.

The predominant sensor was the ACC, possibly because it is one of the most common sensors available on both wearables and mobile devices. In addition, its applications are closely related to energy expenditure and activity recognition tasks, which are very useful in DM problems. Other sensors such as GPS, thermometer, microphone, and ambient light are less commonly used in the reviewed studies. This may be because some of these sensors, such as GPS and microphone, are considered to be more privacy-intrusive by users. Nonetheless, these sensors were shown to be helpful as complements to other sensors for the monitoring of DM-related parameters. Furthermore, it is worth noting that none of the studies used commercial smart insoles but one prototype, especially given the fact that most important complications of DM translate into foot issues.

Diabetes technology has grown in the recent years, with CGM being at the forefront of the devices used. CGM is primarily used to monitor patients with T1D, with increasing use for patients with T2D. However, the use of CGM does not replace the traditional finger-stick test because patients still need to do a meter reading for accuracy, and in most cases, insurance companies do not cover the use of CGM. The fact that mobile and wearable technologies are at the reach of a majority of the population makes the reviewed solutions particularly cost-effective.

The power consumption of the hardware available in both mobiles and wearables has decreased with the latest advances. However, there is still much to be done to reduce it even further. The 5G technology promises to do so by lowering network energy use by almost 90% and increasing battery life, especially for low-power devices [58]. Allowing patients to use wearable devices for prolonged periods without recharging could help foster their use in the diabetic community. The 5G technology will also increase the connection speed and bandwidth in a unit area and will have a very low latency. This will provide higher real-time monitoring capabilities, an increase in the amount of data sent by time unit, and the possibility of having more wearable devices connected to the cloud without requiring the use of smartphones as gateways. All this provides more self-sufficiency to these devices.

Data collected using mobile and wearable devices for continuous monitoring can be mined using AI techniques such as ML. As shown in this review, some authors used ML to extract information from the collected data, where large and heterogeneous data sets generally improve the performance of these techniques. Data from mobile and wearable sensors can, in principle, be used in combination with conventional clinical data to develop more relevant knowledge outcomes. The time and effort required to collect a data set that can be used to apply ML techniques is reduced by the use of mobile and wearable devices. Classical approaches are generally constrained by the number of samples or data points, as these are measured during clinic appointments, thus leading to lengthier collection phases.

The most common ML techniques used in these studies were decision trees and support vector machines, whereas other popular algorithms such as k-nearest neighbors, artificial neural networks, ensemble methods, and deep learning techniques are used less frequently. These techniques, especially when it comes to deep learning, can significantly boost the performance of the results, normally at the expense of having large data sets, a condition that is normally attained when using passive sensing. In general, experimentation with ML algorithms was performed using a small number of methods, whereas the use of a large variety of these techniques normally leads to higher robustness. Very few studies have used complementary clinical data in addition to sensor data, resulting in better models and more relevant outcomes.

Many manuscripts did not mention the endorsement of their studies from an ethics committee or review board. This is especially important because in several cases, people were used to test the proposed solutions. It is even more important to highlight that in very few occasions, the studies described that informed consent was requested from the participants in the trials. This occurs even when sensitive information is recorded from users during monitoring, such as location, video, or call logs. A reason for this could be linked to the rather emerging nature of this field or the lack of realistic clinical studies around which technical solutions have been developed.

Privacy and security issues were weak aspects of the reviewed studies. Researchers should devote more attention to both realizing and explaining proper procedures to ensure that security and privacy are properly addressed in clinical studies. Otherwise, the quality and applicability of the results are compromised. An effort must be made to put into practice the protocols in the trial involving the ethics committee or review boards in the authorization of the studies. Creating proper informed consents forms and using them in the trials should be a major concern for research in this area, especially in lieu of regulations such as the European General Data Protection Regulation. Similarly, information on data management plans can provide further details on how the research has been undertaken.

**Figure 2:**
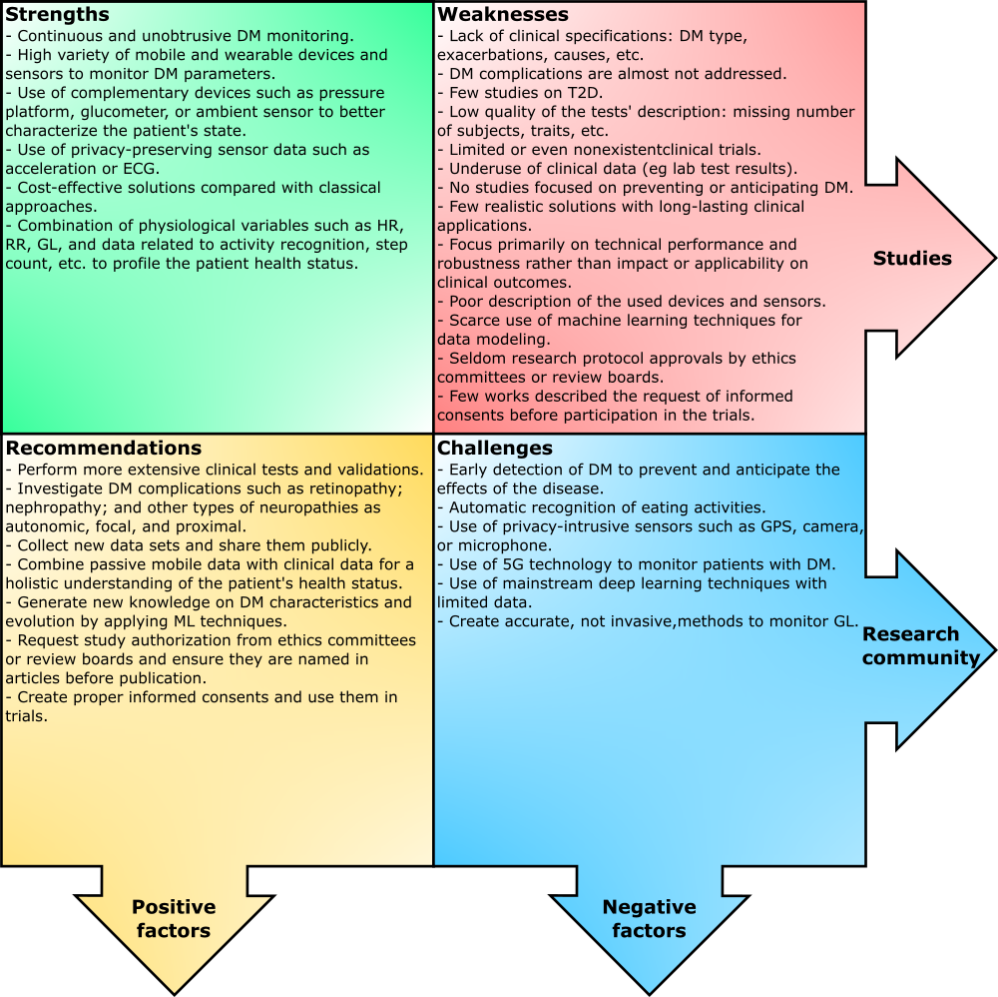
Summary of the principal findings of the reviewed manuscripts. DM: diabetes mellitus; ECG: electrocardiogram; GL: glucose level; HR: heart rate; ML: machine learning; RR: respiration rate; T2D: type 2 diabetes.

A summary of the principal findings described previously is provided in Figure 2. The diagram shows the strengths and weaknesses of the reviewed studies on the monitoring of DM-related parameters as well as the challenges and recommendations for the research community. Strengths are the aspects that these studies have performed well on and could be reproduced in future investigations. Weaknesses are matters that went wrong in these studies and could be improved in future research. Challenges are the elements that the scientific community needs to address successfully to boost the investigation of this topic. Recommendations are the suggestions for the research community working on the monitoring of DM-related parameters.

### Limitations

The monitoring of DM-related parameters using mobile and wearable technology is an emerging field of study. As for any other review, despite having listed a wide variety of terms referring to sensors, wearable devices, and smartphones, new keywords emerge quite often in this rather dynamic technological area, which may have left out some interesting studies from our analysis. Although the search areas of this systematic review (computer science and engineering) are quite large, it is also possible that some relevant studies indexed in other related categories may have been filtered out. We conducted a preliminary check for other domains such as endocrinology metabolism, general internal medicine, or health care sciences services, and we did not find relevant studies that would meet the defined criteria. Other sources of digital data, such as social network interactions, have not been considered in this study, as they can be realized via different technologies besides mobiles and wearables. Nevertheless, it could be interesting to explore the potential of these interactions to explain some relevant behaviors of the patient, such as their mood. The lack of details in some studies also made it difficult to judge whether the authors were using commercial devices or their own prototype. Thus, it is possible that some relevant studies were excluded, although this is in line with the PRISMA guidelines followed in this systematic review.

### Conclusions

As demonstrated in this systematic review, the field of mobile and wearable monitoring of DM-related parameters shows early promise, despite its recent development. Several actors may benefit at the maturation of this field: (1) patients with DM, who may have a better quality of life while improving the management and self-control of the disease or its complications in a continuous, passive, and unobtrusive way; (2) health care professionals and institutions, who may develop the ability to provide medical care and information in a portable and affordable way; and (3) researchers, who may have access to a large and varied amount of data sets to extract relevant information. The aforementioned 3 actors may work in synergy, which motivates a greater and faster evolution of the field. However, some gaps remain to accomplish this view, such as the creation or modification of relevant sensors to be less privacy-intrusive; decreasing the devices’ power consumption; using the advantages of the 5G technology; and, perhaps the most important one, combining passive mobile data with clinical data for a holistic understanding of the patient’s health status. Accomplishing these challenges requires interdisciplinary teams’ collaboration and the appropriate funding of governments and institutions to design and develop the required technologies for sensing the data, designing new and better processing techniques, and creating realistic solutions with long-lasting clinical applications.

## Appendix

Multimedia Appendix 1 Newcastle-Ottawa Scale quality assessment of the selected studies.

Multimedia Appendix 2 Reviewed manuscripts by the year of publication (up to July 28, 2020).

Multimedia Appendix 3 Sensor use for each study.

## Abbreviations

ACC: accelerometer
AI: artificial intelligence
CGM: continuous glucose monitor
DFU: diabetic foot ulcer
DM: diabetes mellitus
DPN: diabetic peripheral neuropathy
ECG: electrocardiogram
FGM: flash glucose monitor
GD: gestational diabetes
GL: glucose level
GM: glucose monitor
HR: heart rate
ML: machine learning
NOS: Newcastle-Ottawa Scale
PRISMA: Preferred Reporting Items for Systematic Reviews and Meta-Analyses
RR: respiration rate
T1D: type 1 diabetes
T2D: type 2 diabetes
WoS: Web of Science

## References

[1] Baena-Díez JM, Peñafiel J, Subirana I, Ramos R, Elosua R, Marín-Ibañez A, Guembe MJ, Rigo F, Tormo-Díaz MJ, Moreno-Iribas C, Cabré JJ, Segura A, García-Lareo M, de la Cámara AG, Lapetra J, Quesada M, Marrugat J, Medrano MJ, Berjón J, Frontera G, Gavrila D, Barricarte A, Basora J, García JM, Pavone NC, Lora-Pablos D, Mayoral E, Franch J, Mata M, Castell C, Frances A, Grau M, FRESCO Investigators (2016). Risk of cause-specific death in individuals with diabetes: a competing risks analysis. Diabetes Care.

[2] (2019). IDF Diabetes Atlas: Ninth Edition 2019.

[3] Zimmet PZ, Magliano DJ, Herman WH, Shaw JE (2014). Diabetes: a 21st century challenge. Lancet Diabetes Endocrinol.

[4] (2017). IDF Diabetes Atlas 8th Ed 2017.

[5] (2019). Classification of Diabetes Mellitus 2019.

[6] American Diabetes Association (2008). Diagnosis and classification of diabetes mellitus. Diabetes Care.

[7] World Health Organization (2009). Mortality and Burden of Disease Attributable to Selected Major Risks.

[8] Contreras I, Vehi J (2018). Artificial intelligence for diabetes management and decision support: literature review. J Med Internet Res.

[9] Alberti KG, Zimmet PZ (1998). Definition, diagnosis and classification of diabetes mellitus and its complications. Part 1: diagnosis and classification of diabetes mellitus. Provisional report of a WHO consultation. Diabet Med.

[10] Diabetes. World Health Organization.

[11] Hu FB, Manson JE, Stampfer MJ, Colditz G, Liu S, Solomon CG, Willett WC (2001). Diet, lifestyle, and the risk of type 2 diabetes mellitus in women. N Engl J Med.

[12] d'Emden MC, Shaw JE, Jones GR, Cheung NW (2015). Guidance concerning the use of glycated haemoglobin (HbA1c) for the diagnosis of diabetes mellitus. Med J Aust.

[13] O'Connor PJ, Bodkin NL, Fradkin J, Glasgow RE, Greenfield S, Gregg E, Kerr EA, Pawlson LG, Selby JV, Sutherland JE, Taylor ML, Wysham CH (2011). Diabetes performance measures: current status and future directions. Diabetes Care.

[14] Cho NH, Shaw JE, Karuranga S, Huang Y, da Rocha Fernandes JD, Ohlrogge AW, Malanda B (2018). IDF Diabetes Atlas: global estimates of diabetes prevalence for 2017 and projections for 2045. Diabetes Res Clin Pract.

[15] American Diabetes Association (2020). 7. Diabetes Technology: standards of medical care in diabetes-2020. Diabetes Care.

[16] Kim BY, Lee J (2017). Smart devices for older adults managing chronic disease: a scoping review. JMIR Mhealth Uhealth.

[17] Demographics of mobile device ownership and adoption in the United States. Pew Research Center.

[18] Global attitudes and trends. Pew Research Center.

[19] Wearables - worldwide. Statista.

[20] Godfrey A, Hetherington V, Shum H, Bonato P, Lovell NH, Stuart S (2018). From A to Z: wearable technology explained. Maturitas.

[21] Richter F (2018). The global wearables market is all about the wrist. Statista.

[22] Felix IR, Castro LA, Rodriguez L, Banos O (2019). Mobile sensing for behavioral research: a component-based approach for rapid deployment of sensing campaigns. Int J Distrib Sens N.

[23] Banos O, Villalonga C, Bang J, Hur T, Kang D, Park S, Huynh-The T, Le-Ba V, Amin MB, Razzaq MA, Khan WA, Hong CS, Lee S (2016). Human behavior analysis by means of multimodal context mining. Sensors (Basel).

[24] Lee U, Han K, Cho H, Chung K, Hong H, Lee S, Noh Y, Park S, Carroll JM (2019). Intelligent positive computing with mobile, wearable, and IoT devices: literature review and research directions. Ad Hoc Networks.

[25] Cahn A, Akirov A, Raz I (2018). Digital health technology and diabetes management. J Diabetes.

[26] Yu K, Beam AL, Kohane IS (2018). Artificial intelligence in healthcare. Nat Biomed Eng.

[27] Jiang F, Jiang Y, Zhi H, Dong Y, Li H, Ma S, Wang Y, Dong Q, Shen H, Wang Y (2017). Artificial intelligence in healthcare: past, present and future. Stroke Vasc Neurol.

[28] Rumbold JM, O'Kane M, Philip N, Pierscionek BK (2020). Big Data and diabetes: the applications of Big Data for diabetes care now and in the future. Diabet Med.

[29] Moher D, Liberati A, Tetzlaff J, Altman DG, PRISMA Group (2009). Preferred reporting items for systematic reviews and meta-analyses: the PRISMA statement. PLoS Med.

[30] Wells GA, Shea B, O'Connell D, Peterson J, Welch V, Losos M, Tugwell P The Newcastle-Ottawa Scale (NOS) for assessing the quality of nonrandomised studies in meta-analyses. Ottawa Hospital Research Institute.

[31] Taskiran NO (2020). Advances in Media, Entertainment, and the Arts (AMEA) book series. Handbook of Research on Multidisciplinary Approaches to Literacy in the Digital Age.

[32] Merickel J, High R, Smith L, Wichman C, Frankel E, Smits K, Drincic A, Desouza C, Gunaratne P, Ebe K, Rizzo M (2019). Driving Safety and Real-Time Glucose Monitoring in Insulin-Dependent Diabetes. Int J Automot Eng.

[33] Rescio G, Leone A, Francioso L, Siciliano P (2019). Smart insole for diabetic foot monitoring. Lecture Notes in Electrical Engineering.

[34] Ramazi R, Perndorfer C, Soriano E, Laurenceau JP, Beheshti R (2019). Multi-modal predictive models of diabetes progression. Proceedings of the 10th ACM International Conference on Bioinformatics, Computational Biology and Health Informatics.

[35] Garcia CA (2019). Non-invasive diabetes detection using facial texture features captured in a less restrictive environment. Int J Recent Technol Eng.

[36] Luštrek M, Cvetković B, Janko V (2014). Monitoring patients’ lifestyle with a smartphone and other devices placed freely on the body. Ambient Intelligence.

[37] Luštrek M, Cvetkovic B, Mirchevska V, Kafalı O, Romero A, Stathis K (2015). Recognising lifestyle activities of diabetic patients with a smartphone. European Union Digital Library.

[38] Calbimonte J, Ranvier J, Dubosson F, Aberer K (2017). Semantic representation and processing of hypoglycemic events derived from wearable sensor data. J Ambient Intell Smart Environ.

[39] Reddy VR, Choudhury AD, Deshpande P, Jayaraman S, Thokala NK, Kaliaperumal V (2017). Dmsense: a non-invasive diabetes mellitus classification system using photoplethysmogram signal. Proceedings of the IEEE International Conference on Pervasive Computing and Communications Workshops (PerCom Workshops).

[40] Bartolic L, Zorić B, Martinović G (2018). E-Gluko: a ubiquitous system for health status monitoring and tracking in diabetes patients. Proceedings of the International Conference on Smart Systems and Technologies (SST).

[41] Gia TN, Dhaou IB, Ali M, Rahmani AM, Westerlund T, Liljeberg P, Tenhunen H (2019). Energy efficient fog-assisted IoT system for monitoring diabetic patients with cardiovascular disease. Future Gener Comput Syst.

[42] Sevil M, Rashid M, Hajizadeh I, Maloney Z, Samadi S, Askari MR, Brandt R, Hobbs N, Park M, Quinn L, Cinar A (2019). Assessing the effects of stress response on glucose variations. Proceedings of the 16th IEEE International Conference on Wearable and Implantable Body Sensor Networks (BSN).

[43] Zherebtsov EA, Zharkikh EV, Kozlov IO, Loktionova YI, Zherebtsova AI, Rafailov IE, Sokolovski SG, Sidorov VV, Dunaev AV, Rafailov EU (2019). Wearable sensor system for multipoint measurements of blood perfusion: pilot studies in patients with diabetes mellitus. Medical Laser Applications and Laser-Tissue Interactions.

[44] Najafi B, Horn D, Marclay S, Crews RT, Wu S, Wrobel JS (2010). Assessing postural control and postural control strategy in diabetes patients using innovative and wearable technology. J Diabetes Sci Technol.

[45] Faccioli S, Ozaslan B, Garcia-Tirado JF, Breton M, Favero SD (2018). Black-box model identification of physical activity in type-1 diabetes patients. Proceedings of the 40th Annual International Conference of the IEEE Engineering in Medicine and Biology Society (EMBC).

[46] McMillan KA, Kirk A, Hewitt A, MacRury S, Lennon M (2018). Methods for combining continuously measured glucose and activity data in people with type 2 diabetes: challenges and solutions. J Rehabil Assist Technol Eng.

[47] Cvetković B, Janko V, Romero AE, Kafalı O, Stathis K, Luštrek Mitja (2016). Activity recognition for diabetic patients using a smartphone. J Med Syst.

[48] Fraiwan L, AlKhodari M, Ninan J, Mustafa B, Saleh A, Ghazal M (2017). Diabetic foot ulcer mobile detection system using smart phone thermal camera: a feasibility study. Biomed Eng Online.

[49] McLean A, Osgood N, Newstead-Angel J, Stanley K, Knowles D, van der Kamp W, Qian W, Dyck R (2017). Building research capacity: results of a feasibility study using a novel mhealth epidemiological data collection system within a gestational diabetes population. Stud Health Technol Inform.

[50] Turksoy K, Monforti C, Park M, Griffith G, Quinn L, Cinar A (2017). Use of wearable sensors and biometric variables in an artificial pancreas system. Sensors (Basel).

[51] Groat D, Kwon HJ, Grando MA, Cook CB, Thompson B (2018). Comparing real-time self-tracking and device-recorded exercise data in subjects with type 1 diabetes. Appl Clin Inform.

[52] Sarda A, Munuswamy S, Sarda S, Subramanian V (2019). Using passive smartphone sensing for improved risk stratification of patients with depression and diabetes: cross-sectional observational study. JMIR Mhealth Uhealth.

[53] Rodríguez-Rodríguez I, Chatzigiannakis I, Rodríguez JV, Maranghi M, Gentili M, Zamora-Izquierdo M (2019). Utility of big data in predicting short-term blood glucose levels in type 1 diabetes mellitus through machine learning techniques. Sensors (Basel).

[54] Sanz AJ, Díez JL, Giménez M, Bondia J (2019). Enhanced accuracy of continuous glucose monitoring during exercise through physical activity tracking integration. Sensors (Basel).

[55] Whelan ME, Orme MW, Kingsnorth AP, Sherar LB, Denton FL, Esliger DW (2019). Examining the use of glucose and physical activity self-monitoring technologies in individuals at moderate to high risk of developing type 2 diabetes: randomized trial. JMIR Mhealth Uhealth.

[56] Grewal GS, Bharara M, Menzies R, Talal TK, Armstrong D, Najafi B (2013). Diabetic peripheral neuropathy and gait: does footwear modify this association?. J Diabetes Sci Technol.

[57] Razjouyan J, Grewal GS, Talal TK, Armstrong DG, Mills JL, Najafi B (2017). Does physiological stress slow down wound healing in patients with diabetes?. J Diabetes Sci Technol.

[58] Agiwal M, Saxena N, Roy A (2017). Ten commandments of emerging 5G networks. Wireless Pers Commun.

