# Supplementary material for: Mobile and Wearable Technology for the Monitoring of Diabetes-Related Parameters: Systematic Review
Source: JMIR Mhealth Uhealth. 2021 Jun 3;9(6):e25138. doi: 10.2196/25138 (PMC8212630; doi:10.2196/25138)
Supplement: Multimedia Appendix 1 [file mhealth_v9i6e25138_app1.pdf]

Multimedia Appendix 1. Newcastle-Ottawa Scale quality assessment of the selected studies.

Articles	Selection	Comparability	Exposure
**COHORT STUDIES**			
Luštrek et al. (2014)[36]	✵		
Luštrek et al. (2015) [37]	✵		
Cvetković et al. (2016) [47]	✵		
Calbimonte et al. (2017)[38]	✵		✵
Fraiwan et al. (2017) [48]	✵		
McLean et al. (2017)[49]	✵		
Razjouyan et al. (2017)[57]	✵		✵✵✵
Turksoy et al. (2017)[50]	✵		✵
Bartolic et al. (2018)[40]	✵		
Faccioli et al. (2018)[45]	✵		✵
Groat et al. (2018)[51]	✵		
McMillan et al. (2018)[46]	✵		
Nguyen Gia et al. (2019)[41]	✵		✵
Rescio et al. (2019)[33]	✵		✵
Sarda et al. (2019)[52]	✵		✵
Ramazi et al., (2019) [34]	✵		✵
Sevil et al., (2019) [42]	✵		✵
Rodriguez-Rodriguez et al., (2019) [53]	✵		✵
Sanz et al., (2019) [54]	✵		✵
**CASE CONTROL STUDIES**			
Grewal et al. (2013) [56]	✵		✵
Merickel et al. (2018)[32]	✵		✵
Zherebtsov et al., (2019) [43]			✵
