# Supplementary figures and images for: Mobile and Wearable Technology for the Monitoring of Diabetes-Related Parameters: Systematic Review
Source: JMIR Mhealth Uhealth. 2021 Jun 3;9(6):e25138. doi: 10.2196/25138 (PMC8212630; doi:10.2196/25138)

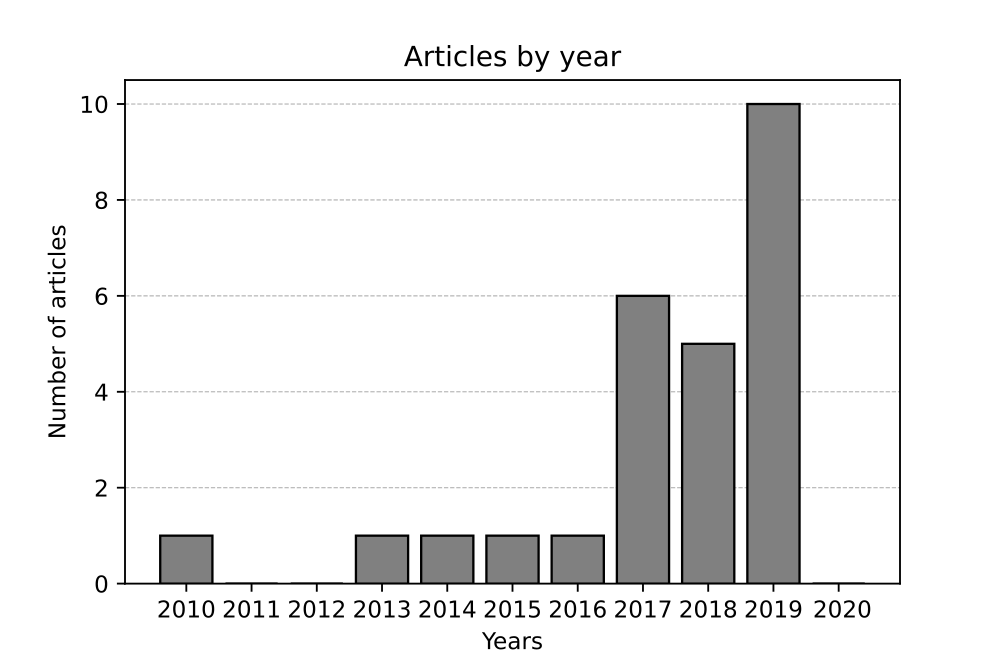

Supplement: Multimedia Appendix 2 [file mhealth_v9i6e25138_app2.png]
