# Supplementary material for: Mobile and Wearable Technology for the Monitoring of Diabetes-Related Parameters: Systematic Review
Source: JMIR Mhealth Uhealth. 2021 Jun 3;9(6):e25138. doi: 10.2196/25138 (PMC8212630; doi:10.2196/25138)
Supplement: Multimedia Appendix 3 [file mhealth_v9i6e25138_app3.pdf]

Multimedia Appendix 3. Sensor use for each study.

Manuscript	ACC	GM	HRM	ECG	Camera	GPS	Thermometer	App	Magnetometer	Wi-Fi	Gyroscope	Galvanometer	Pressure	Microphone	Altimeter	Bluetooth	Oximeter	Inclinometer	OBD	Ambient light	Call logs	Respiration	Humidity	Air quality	Infrared	Heat flux	PPG	Infrared thermopile	Laser Doppler flowmetry	Skin temperature	Total wearable sensors[b]	Total smartphone sensors[b]	Total complementary sensors[b]	Total different sensor types[d]
Turksoy et al. (2017) [50]	W	W	W	W			W					W										C				W					7	0	1	8
Nguyen Gia et al. (2019) [41]	W	W		W			W&C		W		W												C	C							6	0	3	8
McLean et al. (2017) [49]	S				S	S			S	S						S															0	6	0	6
Merickel et al. (2018) [32]	W	W	W		C	C													C												3	0	3	6
Sanz et al., (2019) [54]	W	W&C	W									W			W															W	6	0	1	6
Luštrek et al. (2015) [37]	W&S			W		S				S				S																	2	4	0	5
Cvetković et al. (2016) [47]	W&S			W		S				S				S																	2	4	0	5
Sevil et al., (2019) [42]	W	W										W															W	W			5	0	0	5
Najafi et al. (2010) [44]	W								W		W		C																		3	0	1	4
Bartolic et al. (2018) [40]	W	C	W					S																							2	1	1	4
(Whelan et al., 2019) [55]	W	W	W												W																4	0	0	4
Faccioli et al. (2018) [45]	W	W						S																							2	1	0	3
Groat et al. (2018) [51]		W	W					S																							2	1	0	3
McMillan et al. 2018 [46]	W	W																W													3	0	0	3
Sarda et al. (2019) [52]	S																			S	S										0	3	0	3
Grewal et al. (2013) [56]	W										W																				2	0	0	2
Luštrek et al. (2014) [36]	W&S		W																												2	1	0	2
Calbimonte et al. (2017) [38]	W			W																											2	0	0	2
Fraiwan et al. (2017) [48]					C																				C						0	0	2	2
Razjouyan et al. (2017) [57]	W			W																											2	0	0	2
Reddy et al. (2017) [39]					S												C														0	1	1	2
Rescio et al. (2019) [33]							W						W																		2	0	0	2
Ramazi et al., (2019) [34]	W	W																													2	0	0	2
Zherebtsov et al., (2019) [43]							W																						W		2	0	0	2

Garcia et al., (2019) [35]					S																										0	1	0	1
Rodriguez-Rodriguez et al., (2019) [53]		W																													1	0	0	1
Total per sensor type[a]	19	12	7	6	5	4	4	3	3	3	3	3	2	2	2	1	1	1	1	1	1	1	1	1	1	1	1	1	1	1	22[c]	11[c]	9[c]	3.6[e]

(W) Sensor embedded into a wearable device.
(S) Sensor embedded into a smartphone.
(C) Sensor embedded into a complementary device.
(W&S) Sensor embedded into a wearable device and a smartphone.
(W&C) Sensor embedded into a wearable device and a complementary device.
[a]Number of appearances of each sensor in each study.
[b]Number of sensors embedded into wearable, smartphone, or complementary devices in each study.
[c]Number of studies with at least one sensor embedded into wearable, smartphone, or complementary.
[d]Number of different sensor types used in each study.
[e]Average of different sensor types used per study.
